# Valley fever, mimicker of malignancy

**DOI:** 10.1016/j.heliyon.2023.e12900

**Published:** 2023-01-10

**Authors:** Raffi Gharakhanian, Ai Ohno, Debra Craig, Sharon Wang

**Affiliations:** a Arrowhead Regional Medical Center, Department of Internal Medicine and Infectious Diseases, 400 N Pepper Ave., Colton, CA 92324, USA; b California University of Science and Medicine School of Medicine, 1501 Violet St., Colton, CA 92324, USA

**Keywords:** Disseminated coccidiomycosis, Coccidioides

## Abstract

Coccidioidomycosis rates in endemic areas such as California and Arizona have been increasing in recent years. Most common manifestations in symptomatic individuals involve the lungs. Disseminated disease occurs when the infection spreads beyond the lungs. Disseminated disease occurs in about 1% of all coccidiomycosis cases. Diagnosis in classically non-endemic regions can be difficult as coccidiomycosis can mimic a variety of other illnesses which can lead to delays in initiating appropriate therapy. We report a case of severe disseminated coccidiomycosis involving the soft tissue, bone, and intra-abdominal organs in a previously healthy individual that was initially thought to be a malignancy. With climate change possibly altering the traditional endemic regions and expanding Coccidioides to new territories, this case reinforces the importance of maintaining a broad differential as well as awareness of disease manifestations for healthcare providers who do not regularly treat Coccidioides.

## 1 Introduction

Coccidioidomycosis, commonly known as Valley Fever, is a dimorphic fungal infection that is endemic to the southwestern United States, parts of northern Mexico, and Central and South America [1,2]. Valley Fever is most commonly contracted by inhalation of arthroconidia from disturbed soil resulting in spherule formation in the body which comprises the parasitic phase. Transmission from inanimate objects have been reported in limited cases and are presumed to be due to contact with fomites [3]. Rare cases of human-human and animal-human transmission have been reported within occupational exposure and veterinary medicine requiring manipulation and aerosolization of endospores within diseased tissue [4]. Most individuals exposed to the fungus remain asymptomatic while others may develop flu-like symptoms that last for weeks to months [1]. Yearly reported case numbers of Coccidioides infections have been increasing for several decades to about 20,000 in 2019, with most cases being in the Arizona and California regions [5]. There are approximately 200 yearly Coccidioidomycosis-associated deaths in the United States from the years 1999–2019 [5]. Diagnostic approach is started with serological testing in suspected cases. Enzyme-linked immunoassays (EIA) for IgM/IgG is the most common initial evaluation step due to increased sensitivity and is followed up by an immunodiffusion assay for quantitative analysis to monitor response to therapy [[6], [7], [8]]. Further diagnostic testing can also include histological evaluation for spherules, culturing of body fluid samples, or urinary antigen detection in severely immunocompromised patients when serological testing can be unreliable [6–8]. As Coccidioides is never part of the normal microbiota, identification either through histological or culture directed methods is definitive evidence for infection.

Primary pulmonary disease is the most common initial source of infection with associated low-grade fevers, headache, body aches. Approximately sixty percent of patient are entirely asymptomatic making identification of infected individuals difficult. Radiographic findings on chest X-ray are associated with hilar adenopathy, peribronchial infiltration, or an infiltrate compatible with bronchopneumonia. Several weeks after pulmonary symptoms resolve less than thirty percent of patient can develop tender erythema nodosum lesions over the shins, which is a favorable prognostic sign [9].

Progression to disseminated disease occurs in about one percent of cases and often develops through hematogenous or lymphatic spread and can involve any organ, but involvement of skin, central nervous system, and musculoskeletal system are reported to be most prevalent [10]. Risk factors for dissemination include various immunocompromised states such as HIV as well as reported genetic defects involving interleukin-12/interferon-gamma pathways which are more common in African American, Filipino and Hispanic populations [3,11,12].

## 2 Case

**Fig. 1:**
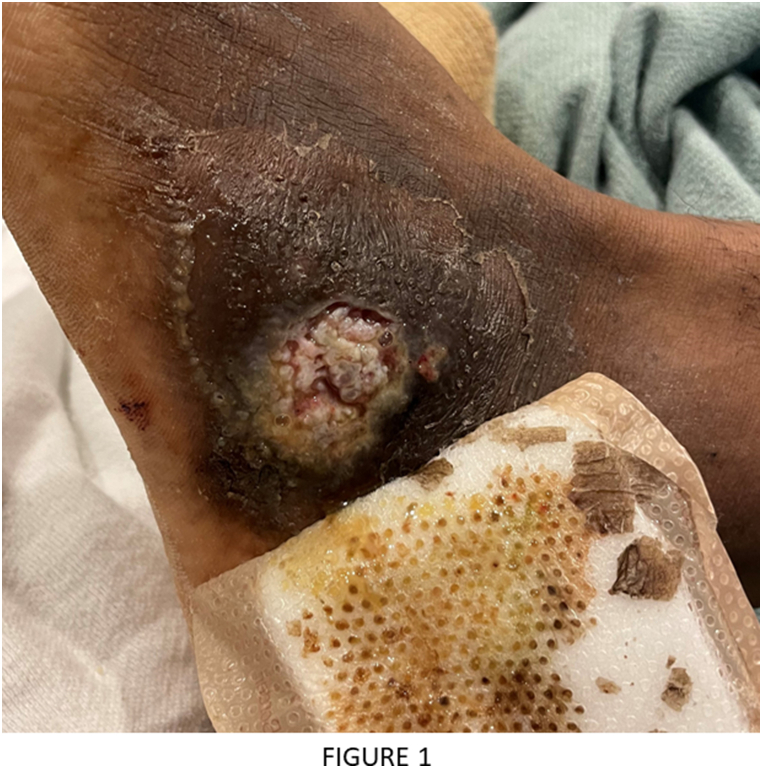
A 3 × 4 cm ulcerating lesion with purulent drainage on the left lower extremity.

**Fig. 2:**
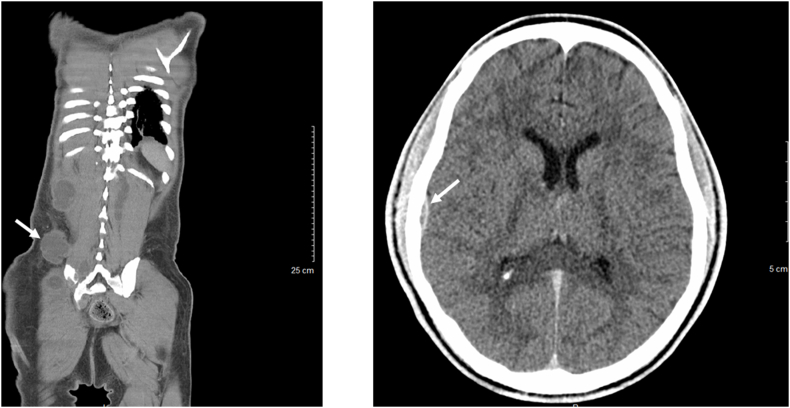
CT radiography of the chest, abdomen, pelvis, head, spine, showing diffuse fluid collections involving the soft tissues and muscles of the abdomen and pelvis, along with focal lytic lesion in the right temporal bone with adjacent focal meningeal thickening, as well as lytic lesions on iliac and sacroiliac regions of the spine.

**Fig. 3:**
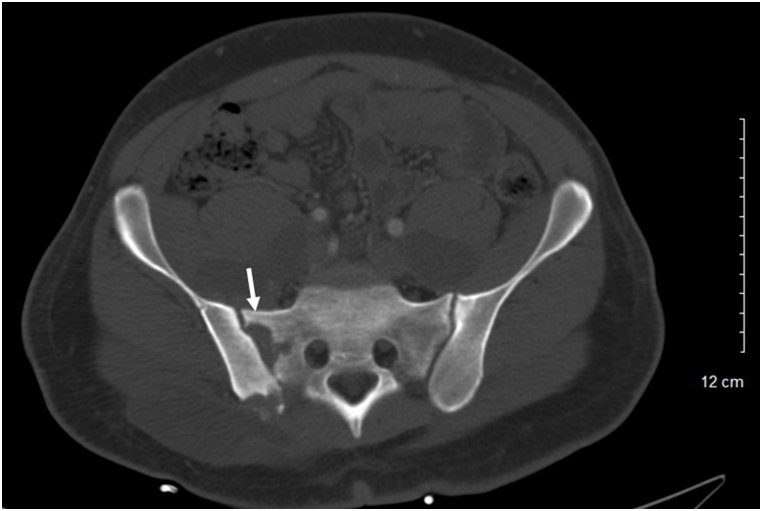
CT radiography of the chest, abdomen, pelvis, head, spine, showing diffuse fluid collections involving the soft tissues and muscles of the abdomen and pelvis, along with focal lytic lesion in the right temporal bone with adjacent focal meningeal thickening, as well as lytic lesions on iliac and sacroiliac regions of the spine.

A 26-year-old previously healthy African American male presented for progressive lower extremity and left shoulder abscesses. Several months prior his presentation he was seen at an outside hospital and given a course of intravenous antibiotics for a presumed bacterial abscess. On admission to our hospital, he complained of continued lower extremity wound drainage along with new abscesses on his left clavicle and multiple lower back soft masses. He endorsed weight loss for 3 months along with decrease in appetite, night sweats, chronic malaise, and generalized discomfort. He denied any history of travel and was born and raised in San Bernardino County, California. On examination, the patient was febrile with tachycardia and had a 1 × 1 cm ulcerating left clavicular lesion, two 5 × 6 cm soft masses on right lower back, and left lower extremity with 3 × 4 cm ulcerating lesion with purulent drainage on dorsal foot (Fig. 1). Complete blood count and basic metabolic panel work up was significant for leukocytosis [∼13^Th^/μL], anemia with hemoglobin [8 g/dL], c-reactive protein level of [33 mg/dL], erythrocyte sedimentation rate of [89 mm/h]. Patient was treated empirically with piperacillin/tazobactam and vancomycin for presumed bacterial infection. Computed topography (CT) of the head, chest, abdomen, pelvis, spine, and lower extremity showed focal lytic lesion in the right temporal bone with adjacent focal meningeal thickening, probable diffuse metastatic disease involving the soft tissues and muscles of the abdomen and pelvis as well as the lumbar, sacral, and Iliac bones (Figs. 2 and 3).

Initial differential diagnosis also included possible testicular malignancy as the patient was not responding to broad empiric antibiotic coverage and complained about vague scrotal pain with associated constitutional symptoms. Malignancy evaluation including a testicular ultrasound, beta-human chorionic gonadotropin and alpha-fetoprotein which were unremarkable, as a result, testicular malignancy moved lower on our differential. Interventional radiology (IR) was consulted for lumbar abscess aspiration for culture and cytology. Infectious disease was consulted for further recommendations given the patient's failure to respond to multiple previous antibiotic regimens.

**Fig. 4:**
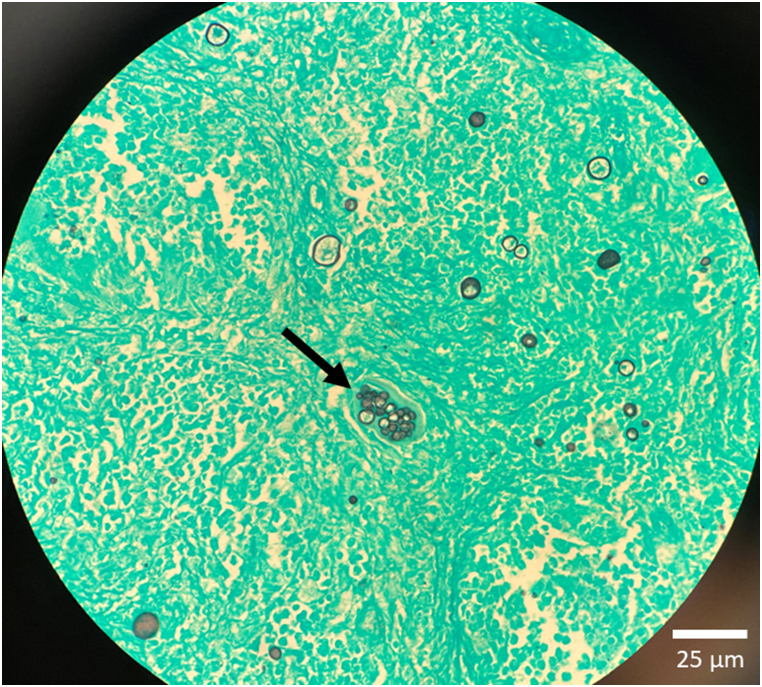
Cytology slide of fluid drained from the lumbar back stained with Grocott's methenamine silver revealed spherules (∼12 μm) consistent with *Coccidioides*.

Cytology slides from the lumbar abscess collection were stained with Grocott's methenamine silver and revealed spherule granules (∼12 μm) consistent with Coccidioides (Fig. 4). Serum Coccidioides complement fixation titer was 1:1024, suggestive of extensive disease burden, as titers >1:16 are generally associated with disseminated disease. These combined findings confirmed our diagnosis of disseminated coccidiomycosis. Lumbar puncture for cerebral spinal fluid analysis was considered, however, patient denied any neurological complaints. Per Infectious Diseases Society of America Coccidioides guidelines, lumbar punctures are not needed in patients without neurological complaints. Patient was started on liposomal amphotericin B [5 mg/kg] and fluconazole [1000 mg daily]. Patient was treated for 19 days inpatient before being discharged with home health nursing. He was planned to continue amphotericin infusions via a peripherally inserted central catheter for five days per week for two weeks followed by three days per week for two months, then transitioned to oral itraconazole. However, he became non-compliant with his treatment and was re-admitted to our hospital with increasing number of lesions after being off anti-fungal therapy for one month. Patient reported developing new lesions at the right lateral malleolus and left neck within one week of stopping treatment. CT radiography of the abdomen and pelvis showed stable multi-focal abscesses throughout intra-abdominal organs and bones. Coccidioides complement fixation titer was unchanged at 1:1024. IR was again consulted to drain the abscesses. Fluid samples were again sent for fungal culture and confirmed to be *Coccidioides immitis* by DNA probe. Patient was treated with liposomal amphotericin for 8 days and subsequently discharged with itraconazole indefinitely. From record review, patient was admitted to multiple other hospitals in the region for similar complaints with repeat aspiration and culture of his abscesses confirming continued coccidioides infection. Patient was ultimately lost to follow up.

Written informed consent was obtained from the patient for publication of this case report and accompanying images.

## 3 Discussion

We report a case of severe disseminated coccidiomycosis involving the soft tissue, bone, and nervous system in an individual with no history of immunodeficiency or risk factors related to spore exposure such a farming or outdoor recreations. Our patient's occupation has been office work for several years. On initial presentation he was suspected to have a metastatic malignancy due to diffuse radiographic lytic like lesions along with severe constitutional symptoms for several months. Cases of disseminated coccidiomycosis masquerading malignancy in the U.S. have previously been reported with patients initially suspected of having bone disease, lymphoma, ovarian cancer, and metastatic disease [[13], [14], [15], [16]]. Of these cases, one had a similar presentation to our patient and involved a 44-year-old African American male with no past medical history presenting with lesions over his left clavicle and scapula for three months. In our patient, diagnosis was complicated by his lack of clear risk factors and pulmonary manifestations, as well as vague symptoms suggestive of malignancy.

Diagnosis in classically non-endemic regions can be difficult as the flu-like symptoms of Valley Fever can mimic a variety of other respiratory illnesses. Even if coccidioidomycosis is suspected, confirmatory testing may take up to two weeks for the antibodies to be detected on serology or the fungus to grow on culture. These tests are also less likely to be ordered in an outpatient setting (e.g., urgent care, emergency department) at the time of initial encounter. These combined factors can lead to delays in initiating appropriate therapy until the disease has progressed in severity.

Coccidioidomycosis rates in endemic areas have been increasing in recent years and cases are expected to double or triple in the coming decades in response to climate change [1,17,18]. The Coccidioides species are dimorphic fungi that grow as hyphae in desert soils. They proliferate during wet, rainy seasons and the hyphae later breaks apart into spore containing fragments when the soil becomes dry. These spores become airborne with any type of soil disturbance (e.g., high winds, earthquakes). As such, the prevalence of Coccidioides species is heavily influenced by environmental conditions and is common in the regions that are hot and dry. With global warming and changes in rain patterns, Coccidioides is expected to expand north into drier states such as Idaho, Wyoming, Montana, Nebraska, and North and South Dakota [17,19]. As such, healthcare providers in these traditionally non-endemic areas should anticipate an increase in Valley fever cases and include the disease as part of their differential diagnoses. Widespread disease can be mistaken for metastatic malignancy, especially with imaging suggestive of diffuse or focal neoplastic skeletal involvement. This case report highlights the importance of obtaining tissue culture and histology in patients presenting with cutaneous lesions unresponsive to antibiotic therapy, even if there is no history of residence or travel to classically endemic regions.

## Author contribution statement

All authors listed have significantly contributed to the investigation, development and writing of this article.

## Funding statement

This research did not receive any specific grant from funding agencies in the public, commercial, or not-for-profit sectors.

## Data availability statement

Data included in article/supp. material/referenced in article.

## Declaration of interest's statement

The authors declare that they have no known competing financial interests or personal relationships that could have appeared to influence the work reported in this paper.
